# Documento de Posición Oficial sobre la Promoción Global de Cuidados Paliativos: Recomendaciones del Grupo Internacional Asesor PAL-LIFE de la Academia Pontificia de la Vida, Ciudad del Vaticano

**DOI:** 10.1089/jpm.2018.0387

**Published:** 2018-10-09

**Authors:** Carlos Centeno, Thomas Sitte, Liliana de Lima, Sami Alsirafy, Eduardo Bruera, Mary Callaway, Kathleen Foley, Emmanuel Luyirika, Daniela Mosoiu, Katherine Pettus, Christina Puchalski, M.R. Rajagopal, Julianna Yong, Eduardo Garralda, John Y. Rhee, Nunziata Comoretto

**Affiliations:** ^1^ PAL-LIFE Project—Pontifical Academy for Life, International Advisory Group for Global Palliative Care Advocacy, Vatican City.; ^2^ ATLANTES Research Programme, Institute for Culture and Society, University of Navarra, Pamplona, Spain.; ^3^ Deutsche PalliativStiftung, Fulda, Germany.; ^4^ International Association for Hospice and Palliative Care (IAHPC), Houston, Texas.; ^5^ Palliative Medicine Unit, Kasr Al-Ainy School of Medicine, Cairo University, Cairo, Egypt.; ^6^ Department of Palliative Medicine and Supportive Care, UT MD Anderson Cancer Center, Houston, Texas.; ^7^ Memorial Sloan Kettering Cancer Center, New York, New York.; ^8^ African Palliative Care Association, Kampala, Uganda.; ^9^ Casa Sperantei, Transylvania University, Brasov, Romania.; ^10^ The George Washington University's Institute for Spirituality and Health (GWish), Washington, District of Columbia.; ^11^ WHO Collaborating Centre for Training and Policy on Access to Pain Relief, Pallium India, Trivandrum, Kerala, India.; ^12^ The Catholic University of Korea (CUK), Seoul, Korea.; ^13^ Icahn School of Medicine at Mount Sinai, New York, New York.; ^14^ Pontifical Academy for Life, Vatican City.

**Keywords:** Cuidados Paliativos, desarrollo, promoción, global, posición oficial

## Abstract

***Contexto:*** La Academia Pontificia de la Vida (PAV) es una institución académica de la Santa Sede (Vaticano) cuyo objetivo es promover una visión católica de la ética biomédica. La PAV invitó a una serie de expertos en Cuidados Paliativos (CP) de todo el mundo, de todas las creencias, a desarrollar recomendaciones estratégicas para el desarrollo global de CP (“Grupo PAL-LIFE”).

***Diseño:*** Trece expertos internacionales reconocidos por su actividad promotora global de CP participaron en un estudio Delphi on-line. En un proceso de cuatro rondas, se pidió a los participantes que identificasen los grupos de interés o instituciones claves para la promoción de CP y que propusieran, para cada uno de ellos, recomendaciones estratégicas para el desarrollo de CP. Cada ronda incorporaba los comentarios de las rondas previas hasta lograr el consenso en las recomendaciones más importantes. En una última fase, al grupo de expertos se le solicitó la jerarquización por importancia de los grupos clave en una escala de 1 a 13. También se solicitaron sugerencias concretas para la implementación de las recomendaciones. Mediante análisis clúster se ordenaron los grupos de interés en dos niveles de importancia para el desarrollo de CP.

***Resultados:*** Trece recomendaciones fueron seleccionadas como las más importantes (una por cada grupo clave). Las recomendaciones para los grupos mejor puntuados fueron: (1) Responsables Políticos: garantizar el acceso universal a los CP; (2) Academia: ofrecer cursos obligatorios de CP en el pregrado; (3) Profesionales sanitarios: promover una certificación adecuada; (4) Hospitales e Instituciones sanitarias: asegurar el acceso a medicamentos de CP; y (5) Asociaciones de CP: ser promotoras eficaces y trabajar con los gobiernos en la implementación de las recomendaciones internacionales sobre CP. También se presentan recomendaciones para los ocho grupos clave restantes.

***Discusión:*** Este documento representa la posición oficial de la PAV en lo que respecta a estrategias de promoción para el desarrollo de los CP en el mundo.

## Contexto

Cada año, más de 25 millones de personas fallecen con un sufrimiento grave relacionado con la salud (SGS) con enfermedades limitantes y que comprometen la vida. Otros 35 millones de personas no fallecen pero viven con similares condiciones y SGS^1^. Aún hoy la inmensa mayoría de la población mundial no tiene acceso a los tratamientos, la asistencia y el apoyo social adecuado en esas circunstancias.

Los cuidados paliativos (CP) ayudan a aliviar el SGS proporcionando cuidados físicos, psicosociales y espirituales tanto a los pacientes como a sus familias. Los CP alivian el “dolor total” cambiando desde el modelo habitual médico sobre-tecnificado a un modelo holístico, centrado en la persona^2^.

Se estima que las necesidades no cubiertas de cuidados paliativos a nivel mundial afectan a 27 millones de personas cada año^3^. Para otros trabajos la necesidad es incluso superior, de hasta 40 millones de personas cada año^4^, existiendo una prevalencia de SGS que afecta a 61 millones de personas^1^. Diversos estudios han presentado el deficit de la provisión de CP con respecto a su demanda^5–9^, y señalan la falta de acceso a CP como un problema global de desigualdad sanitaria^10,11^.

También se constata en todo el mundo un aumento de la carga de enfermedades no transmisibles (ENT). Globalmente, las ENT provocan un 70% de todas las muertes^12^ y generan un 93% de las necesidades de CP en población adulta. Casi un 80% de esa necesidad global de CP se circunscribe a países con un nivel de ingresos medio-bajo^3,5^. Además, la población mundial está envejeciendo, y ésto, junto con la creciente prevalencia de ENT y la persistencia de otras enfermedades crónicas e infecciosas, anuncia una subida alarmante en la necesidad de CP a nivel global^4^. De hecho, hay estudios que estiman que para el año 2040, la proporción de habitantes que necesitarán CP a nivel mundial va a incrementarse desde el 25% al 47%^13^.

Esta necesidad creciente es reconocida por las organizaciones internacionales de salud. La Organización Mundial de la Salud (OMS) aprobó recientemente el XIII Programa General de Trabajo en que admitía la limitada disponibilidad de servicios de CP en gran parte del mundo y la existencia de enormes sufrimientos evitables por este motivo para millones de pacientes y sus familiares”^4,14^ y concluía con varias recomendaciones para un mayor desarrollo de CP y solicitando más apoyo para campañas de promoción global de CP. Aunque los estudios de investigación señalan que hay un crecimiento sostenido de los cuidados paliativos a nivel global, la demanda desborda la provisión, y además el crecimiento ha sido muy desigual, con muchos países que han progresado muy poco en la última década^4–9^.

El aprecio de la Iglesia Católica por los CP como un modo de cuidar a los más vulnerables es evidente en su catecismo, que incluye la siguiente declaración “Los cuidados paliativos constituyen una forma especial de caridad desinteresada, y por esta razón deben ser alentados” (Catecismo de la Iglesia Católica, n. 2279). Recientemente, el Papa Francisco pronunció unas sentidas palabras sobre los CP dedicadas a los profesionales de la salud: “Animo a los profesionales y a los estudiantes a especializarse en este tipo de asistencia, que no es menos valiosa por el hecho de no salvar vidas. Los [CP] logran algo igualmente importante: valoran a la persona”^15^.

La Academia Pontificia de la Vida (PAV) es una institución académica de la Santa Sede (Vaticano) dedicada a la promoción de la vida humana, -que entre otros temas específicos-, investiga los aspectos relacionados con la ética médica. En 2017, la PAV puso en marcha un proyecto internacional llamado: “PAL-LIFE: Un Grupo de Trabajo Asesor Internacional sobre la difusión y desarrollo de cuidados paliativos en el mundo”, que tiene como fin aconsejar sobre cómo la Iglesia Católica puede ayudar en el desarrollo continuado de los CP en el mundo^16^. Este documento representa la postura oficial de la PAV sobre CP, y se pretende que sea empleado en la defensa y promoción de los CP con los gobiernos locales, las organizaciones sanitarias, líderes locales y grupos religiosos.

## Diseño

Se desarrolló un proceso de consenso entre 13 expertos en desarrollo global de CP para generar recomendaciones para los principales grupos de interés, incluyendo un ranking por nivel de importancia, tanto de las recomendaciones como de los grupos, así como sugerencias para su puesta en acción.

El estudio fue sometido y aprobado por el Comité Ético de Investigación Clínica de la Universidad de Navarra.

### Selección de expertos y definición del proceso

El grupo de expertos en desarrollo global de CP se seleccionó en base a criterios de representatividad geográfica y profesional. Entre los miembros figuran perfiles clínicos, eticistas, y administradores sanitarios que trabajan en centros académicos u organizaciones nacionales e internacionales de CP, y que profesan distintas creencias religiosas. Inicialmente, la PAV escogió tres expertos en la promoción de CP con experiencia en el desarrollo global de CP. En un segundo paso, se añadieron otros expertos al grupo a través un proceso de “bola de nieve” con recomendaciones por pares de al menos dos expertos, así hasta alcanzar un total de 13 miembros (grupo ad hoc), número que pareció suficiente. En la última fase del proceso intervinieron dos expertos más por recomendación de varios miembros del grupo. La tabla 1 muestra los miembros del grupo ad hoc.

**Tabla 1.** Miembros Del Grupo PAL-LIFE

*Nombre*	*Título, Institución*	*Ciudad*	*País*
Samy Alsirafy	Jefe de la Unidad de Medicina Paliativa, Escuela de Medicina Kasr Al-Ainy, Universidad de El Cairo	El Cairo	Egipto
Eduardo Bruera	Jefe del Departamento de Medicina Paliativa y Cuidados de Soporte, UT MD Anderson Cancer Center	Houston	EEUU
Mary V. Callaway	Comité directivo, Asociación Internacional de Hospice y Cuidados Paliativos (IAHPC)	Houston	EEUU
Carlos Centeno	Investigador Principal del Grupo de Investigación Atlantes, Universidad de Navarra	Pamplona	España
Liliana De Lima	Directora Ejecutiva, IAHPC	Houston	EEUU
Kathleen M Foley	Neuróloga, Memorial Sloan Kettering Cancer Center	Nueva York	EEUU
Emmanuel Luyirika	Director Ejecutivo, Asociación Africana de Cuidados Paliativos (APCA)	Kampala	Uganda
Daniela Mosoiu	Directora, Hospice Casa Sperantei, Profesora Asociada de la Universidad de Transylvania	Brasov	Rumania
Katherine Pettus	Portavoz Oficial, IAHPC	Houston	EEUU
Christina Puchalski	Directora, Instituto de Espiritualidad y Salud de la Universidad George Washington (GWish), Profesora de Medicina GWU	Washington	EEUU
MR Rajagopal	Director Pallium India, Centro Colaborador de la OMS para la educación y políticas sobre acceso al alivio del dolor	Trivandrum	India
Thomas Sitte	CEO Deutsche PalliativStiftung	Fulda	Alemania
Jin-Sun Yong	Directora, Universidad Católica de Corea (CUK), Centro Colaborador de la OMS para la educación en Hospice y Cuidados Paliativos. Profesora de enfermería, CUK	Seúl	Corea del Sur

La reunión inicial del grupo tuvo lugar en la sede de la PAV en Roma, en marzo de 2017. El objetivo de la reunión era definir la estrategia y decidir la metodología del grupo ad hoc para identificar las recomendaciones. La primera versión del proyecto se redactó ya como un borrador de documento de posición oficial sobre la promoción de los CP, que contendría un compendio de recomendaciones para la planificación en políticas sanitarias y que sirviera de guía y referencia a distintos grupos de interés claves en el desarrollo nacional y regional de CP.

Para los propósitos de este proyecto, el grupo ad hoc ha utilizado la definición de CP de la OMS y ha adoptado su marco estratégico de salud pública para la integración de CP^17^.

### Identificación de grupos de actores clave

Los expertos del grupo ad hoc fueron invitados por correo electrónico a identificar los grupos más relevantes de interés a los que dirigir recomendaciones. Estos grupos de actores clave fueron identificados considerando el rol fundamental que ocupan en la promoción del desarrollo de CP a nivel regional o nacional, en el ámbito sanitario o en la sociedad. A partir de la lista inicial, en la primera ronda de un proceso Delphi de consenso, los miembros del grupo ad hoc propusieron nuevos grupos de interés o modificaron los existentes en la lista, hasta un total de 13 grupos. En base a su área de experiencia, a cada experto se le asignó un grupo específico de interés. La tabla 2 muestra los grupos de interés elegidos.

**Tabla 2.** Ranking de Grupos de Interés para el desarrollo de Cuidados Paliativos

*Grupo de actores clave*	*Puntos*	*Grupo K-mean*
Responsables políticos	122	103.4
Universidades (academia)	111	
Trabajadores Sanitarios	103	
Hospitales y Centros de Salud	92	
Asociaciones de Cuidados Paliativos	89	
Organizaciones Internacionales	71	52.4
Medios de Comunicación	69	
Organizaciones filantrópicas y de caridad	62	
Autoridades Farmacéuticas	59	
Pacientes y Grupos de Pacientes	53	
Profesionales del Cuidado Espiritual	50	
Asociaciones profesionales ajenas a los cuidados paliativos	29	
Farmacéuticos	26	

Puntuaciones en importancia relativa (rango de 1 a 156) y grupo k-means para el análisis clúster.

### Proceso de consenso para las recomendaciones

En la segunda ronda, cada miembro fue contactado de nuevo por correo electrónico, y se le solicitaron dos o tres recomendaciones para su correspondiente grupo de actores clave. Cada recomendación se acompañó de una justificación, con sus referencias bibliográficas, con un límite aproximado de 200 palabras. Las recomendaciones se construyeron teniendo en cuenta publicaciones previas al respecto y la experiencia de los expertos en sus propias instituciones o en otras distintas. Esto permitiría obtener las mejores recomendaciones posibles por cada grupo clave de interés.

En la tercera ronda, todas las recomendaciones fueron compartidas con el grupo ad hoc al completo a través de una herramienta de encuestas on-line (https://es.surveymonkey.com/) y a cada miembro del grupo ad hoc se le pidió que puntuase cada recomendación en una escala *likert* de 1 a 5 (siendo 1 “para nada importante” y 5 “extremadamente importante”). Se calcularon posteriormente las puntuaciones medias otorgadas a cada recomendación.

Los resultados se presentaron preliminarmente en una conferencia de CP organizada por la PAV en Roma en marzo de 2018, y posteriormente se discutieron en una nueva reunión cara a cara por una parte del grupo de expertos. En la reunión, se llevó a cabo una revisión de texto y se sugirió mejorar la formulación.

En una cuarta ronda, doce miembros del grupo ad hoc revisaron sus puntuaciones previas y realizaron una nueva puntuación de los grupos de interés en base a la percepción de su importancia para el desarrollo de CP. Utilizando una escala de 13 puntos, se asignaron puntuaciones a cada grupo, conforme al ranking dado por cada miembro [rango: desde 12 (peor = 1 punto por experto) a 156 (mejor = 13 puntos por experto)]. Un análisis exploratorio de tipo clúster facilitó una clasificación de los grupos de interés en dos niveles distintos de importancia para el desarrollo de CP. Como último paso, también en la cuarta ronda, se pidió a los miembros del grupo ad hoc que propusieran sugerencias de implementación que ayudaran a llevar a cabo cada una de las recomendaciones.

### Aprobación y respaldo de la PAV de las recomendaciones y presentación de resultados

Las recomendaciones resultantes de cada grupo de interés fueron revisadas y aceptadas por el grupo, y posteriormente recibieron la aprobación y el respaldo del el Comité Directivo de la PAV. Este apoyo se anunció durante la sesión plenaria del encuentro anual de la PAV (junio de 2018) como la posición oficial de la Academia y las recomendaciones de PAL-LIFE.

En este artículo presentamos el grupo de cinco recomendaciones más importantes (“de primera línea”) con sugerencias concretas para su implementación. Las ocho recomendaciones restantes para otros grupos de interés se presentan como intervenciones de segunda línea sin detalle de sugerencias en este caso. Todas las recomendaciones van acompañadas de una justificación con sus referencias bibliográficas. Las recomendaciones discutidas por el grupo pero que no obtuvieron puntuaciones altas, se han incluido en un informe más extenso disponible en el sitio web de la PAV (www.academyforlife.va).

## Resultados

El resultado de la primera ronda fue la identificación de 13 grupos de interés y la propuesta de 43 recomendaciones. Del total, se seleccionaron 13 recomendaciones como más importantes (una por cada grupo de interés) y son presentadas aquí. Éstas 13 recomendaciones y las otras 30 restantes, están disponibles en el sitio web de la PAV.

La tabla 2 indica la lista de grupos de interés y las puntuaciones en el ranking de importancia para el desarrollo de CP (rango 1–156). El análisis clúster K-means confirmó la existencia de dos niveles en el ranking de grupos de interés: cinco grupos obtuvieron puntuaciones más altas (más cercanas a K-mean 103.4) y nueve grupos obtuvieron puntuaciones más bajas (más cercanas a K-mean 52.4).

Los grupos de interés de primer nivel para la promoción de CP fueron: (1) responsables políticos (en todos los niveles de gobierno); (2) academia (universidades y facultades); (3) trabajadores de la salud; (4) hospitales y centros de salud; y (5) asociaciones profesionales de CP. Las recomendaciones para el primer nivel de grupos de interés, junto a las sugerencias de implementación, se presentan en la tabla 3.

**Tabla 3.** Recomendaciones Del Grupo PAL-LIFE Para Los Principales Grupos de Interés en la Promoción de Cuidados Paliativos, y Sugerencias Para Su Implementación

*Posición*	*Grupo de interés y sugerencia*
1	**Responsables Políticos:** Los responsables políticos deben reconocer el valor social y ético de los Cuidados Paliativos y modificar las estructuras sanitarias, sus políticas y los modos actuales de medir los resultados, para garantizar un acceso universal a los CP para todos aquellos que los necesitan. Deberían también tomar medidas para asegurar un sistema sanitario integrado, que permita un flujo continuo de pacientes entre los distintos niveles de atención, para que los enfermos con problemas complejos puedan ser derivados a niveles secundarios y terciarios, según su necesidad, y derivados de vuelta al domicilio, cuando sea posible.
	Sugerencias para la implementación:
	− Involucrar a las asociaciones nacionales para que promuevan los cuidados paliativos
	− Defender con responsables políticos locales el acceso a los cuidados paliativos como un derecho humano
	− Establecer vínculos con otras iniciativas como el movimiento por el cuidado integral u holístico de la persona, la medicina preventiva y los programas de promoción de salud
	− Realizar una campaña de concienciación pública centrada en el sufrimiento inútil y en la responsabilidad ética del gobierno
	− Incluir cuidados paliativos como componente del plan nacional de enfermedades no transmisibles
2	**Academia (universidades y facultades):** Todas las instituciones académicas que ofrecen grados de salud deberían incluir cursos obligatorios de cuidados paliativos como parte del currículo de pregrado.
	Sugerencias para la implementación:
	− Aprobar una ley nacional que establezca como obligatoria la enseñanza de cuidados paliativos en los grados de salud
	− Desarrollar un currículo estándar para enseñar cuidados paliativos basados en equipos inter-disciplinarios
	− El currículo de cuidados paliativos debe combinar componentes teóricos y prácticos integrados en la atención primaria
	− Cuidados Paliativos debería enseñarse por Profesores con experiencia clínica y nombramiento académico
	− La financiación de los programas educativos de paliativos debe proceder de los presupuestos del gobierno para educación sanitaria
	− Donde aún no se enseñe cuidados paliativos, se puede invitar a dar clases a profesionales expertos de cuidados paliativos, y así crear demanda
	− En Europa y en otros países, se puede adoptar y utilizar el documento de “Recomendaciones de la EAPC sobre cuidados paliativos en el pregrado de facultades de medicina y enfermería”. Para países de Latinoamérica se puede usar el proyecto “Introducción a la Transformación del Sistema” (Initiation for System Transformation Project) (ITES)
	− Promover cursos de “formación de formadores”, también para educación en atención primaria
3	**Trabajadores Sanitarios:** Los profesionales sanitarios que trabajan en cuidados paliativos deben recibir una certificación apropiada, y también deben participar activamente en cursos de formación continua para mantener un nivel de competencia adecuado.
	Sugerencias para la implementación:
	− Para lograr el reconocimiento de cuidados paliativos como una especialidad, las asociaciones nacionales de paliativos deberían trabajar con las Organizaciones Nacionales de los médicos y de las enfermeras (asociaciones colegiales) y con los Ministerios de Sanidad y Educación
	− En cada país se puede establecer un grupo de trabajo entre las asociaciones colegiales de médicos y enfermeras con expertos de cuidados paliativos, para determinar el nivel mínimo de competencias, conocimientos y habilidades y el número de años de dedicación requeridos para obtener el reconocimiento o capacitación de profesional de cuidados paliativos
	− Estandarizar la formación de los profesionales mediante programas de certificación básica y especializada siguiendo el mismo proceso de certificación oficial de profesionales de la salud de cada país
4	**Hospitales y Centros de Salud:** Todos los hospitales y centros de salud deben garantizar un acceso rápido a los medicamentos de cuidados paliativos incluidos en la Lista Modelo de Medicamentos Esenciales de la OMS, particularmente, a la morfina de liberación inmediata. Así mismo deben aceptar en su estructura la provisión de servicios de cuidados paliativos como un imperativo ético y moral.
	Sugerencias para la implementación:
	− Garantizar la formación de todo el personal en fundamentos de cuidados paliativos
	− Definir una estrategia de integración de cuidados paliativos para el hospital o centro de salud
	− Establecer en cada centro un conjunto mínimo de datos para monitorizar la calidad del cuidado en la enfermedad avanzada y al final de la vida.
5.	**Asociaciones de Cuidados Paliativos:** Los representantes de las Asociaciones Nacionales de Cuidados Paliativos deben ser promotores eficaces y trabajar con sus gobiernos en el proceso de implementación del marco político internacional, incluyendo las convenciones, resoluciones y declaraciones en sus respectivos países (ej.: documento resultante de la Asamblea General de las Naciones Unidas (UNGASS), Agenda 2030, Resolución de la Asamblea Mundial de la Salud (WHA).
	Sugerencias para la implementación:
	− Organizar talleres de promoción con representantes de asociaciones nacionales para potenciar a los representantes de la sociedad civil, y que éstos a su vez adopten las habilidades necesarias para una promoción eficaz en campañas y estrategias
	− Las asociaciones nacionales tienen el poder y la legitimidad para requerir a sus gobiernos la implementación de las políticas y acuerdos internacionales sobre cuidados paliativos a nivel nacional, el fortalecimiento de los programas de enfermedades no transmisibles y la adopción de los objetivos del desarrollo sostenible de la agenda 2030.
	− Trabajar para establecer estándares en cuidados paliativos: formación en cuidados paliativos en atención primaria y especializada, desarrollar una estrategia nacional de cuidados paliativos integrada en la atención sanitaria universal, trabajando tanto con organizaciones gubernamentales como no gubernamentales

El segundo nivel de grupos de interés (tabla 4) son los siguientes: (6) organizaciones internacionales; (7) los medios de comunicación; (8) organizaciones filantrópicas y de caridad; (9) autoridades farmacéuticas; (10) pacientes y grupos de pacientes; (11) profesionales del cuidado espiritual; (12) otras sociedades y asociaciones profesionales distintas de CP; y (13) farmacéuticos.

**Tabla 4.** Recomendaciones Del Grupo PAL-LIFE Para el Segundo Nivel de Grupos de Interés Para la Promoción de Cuidados Paliativos

*N*	*Recomendaciones*
6	**A Organizaciones Internacionales:** Las organizaciones internacionales deben animar a los Estados Miembros de la OMS a desarrollar políticas y procedimientos para implementar la resolución 67/19 de la WHA como una parte integral de sus estrategias, y a implementar la Agenda 2030 para cumplir los Objetivos de Desarrollo Sostenible, poniendo especial empeño en las necesidades de los niños y las personas mayores.
7	**A Medios de Comunicación:** Los medios de comunicación deben involucrarse en la creación de una cultura que entienda sobre la enfermedad avanzada y el rol de los cuidados paliativos a lo largo de la vida y como un componente de la Cobertura Sanitaria Universal (UHC).
8	**A Organizaciones Filantrópicas y de Caridad:** Los individuos y organizaciones involucradas con los cuidados paliativos deben atraer, promover y formar para que las organizaciones filantrópicas y de caridad apoyen el desarrollo de cuidados paliativos y la organización de servicios.
9	**A Autoridades Farmacéuticas:** La morfina (preferiblemente en su formulación oral de liberación inmediata) es el medicamento ideal para el tratamiento del dolor por cáncer moderado/severo y para cuidados paliativos, y debería estar disponible y accesible. Ningún gobierno debería aprobar formulaciones de morfina de liberación controlada, parches de fentanilo transdérmico, u oxicodona de liberación prolongada sin garantizar al mismo tiempo la disponibilidad general de la morfina oral de liberación inmediata.
10	**A Pacientes y Grupos de Pacientes:** Los pacientes y los grupos de pacientes pueden ser de gran ayuda en el desarrollo y solicitud de una campaña de educación sanitaria para todos los pacientes con necesidades paliativas y sus familiares, para que éstos incrementen su conocimiento y entendimiento sobre los cuidados paliativos y potencien su rol en el proceso de toma de decisiones.
11	**A los Profesionales del Cuidado Espiritual:** Las instituciones religiosas y grupos de cuidado espiritual deben trabajar para integrar los cuidados espirituales –incluida la evaluación permanente del distrés y el bienestar espiritual- en las guías asistenciales y como un componente de la provisión rutinaria de cuidados paliativos.
12	**A otras Asociaciones y Sociedades Profesionales que no son de Cuidados Paliativos:** Las asociaciones y sociedades profesionales deben alentar a que las organizaciones de derechos humanos asuman las declaraciones existentes, y que implementen estrategias cuyo objetivo sea la mejora del desarrollo de cuidados paliativos en el mundo dentro del marco de los derechos humanos.
13	**A Farmacéuticos:** Los farmacéuticos deben jugar un papel activo en los equipos de cuidados paliativos en la evaluación de la adecuación de los medicamentos prescritos a los pacientes, en la administración puntual, en la educación de los miembros del equipo sobre las interacciones farmacológicas, y la comprensión de pacientes y cuidadores del régimen prescrito para asegurar una correcta adherencia al tratamiento.

### Reflexiones para los grupos de actores clave de primer nivel en la promoción de CP

#### A los responsables políticos

Los pacientes con cáncer o enfermedades crónicas progresivas como insuficiencia cardiaca congestiva, enfermedad pulmonar obstructiva crónica, y VIH-SIDA, presentan graves síntomas físicos, psicosociales y espirituales antes de fallecer^1,18^. Hay evidencia de que los CP reducen gran parte de ese sufrimiento en los pacientes, así como el estrés psicosocial, espiritual o existencial en las familias^19^.

También hay fuerte evidencia de que estos beneficios van acompañados de una reducción del coste total de la atención^20^. Este ahorro se consigue fundamentalmente al evitar investigaciones y tratamientos inútiles centrados en la enfermedad base y también hospitalizaciones en centros de agudos y unidades de cuidados intensivos^21–25^. El valor añadido en los cuidados de salud resulta de un balance adecuado entre beneficios y costes. Los CP han demostrado tener impacto positivo en ambos componentes de ese valor.

#### A la academia (universidades y facultades)

De acuerdo al Comité de Derechos Económicos, Sociales y Culturales de las Naciones Unidas (CESCR), los Estados Miembros están obligados a garantizar el acceso universal a los CP. Esta obligación incluye la tarea de asegurar que los profesionales sanitarios alcancen un nivel educativo suficiente^4^. Del mismo modo, la OMS urge a los Estados Miembros a integrar la educación básica en CP en el pregrado de todos los futuros profesionales de medicina y enfermería^11^. En otras palabras, las leyes internacionales estipulan que los gobiernos y universidades de los Estados Miembros provean formación adecuada a los trabajadores sanitarios con arreglo a los principios establecidos por la OMS^26^.

Diversos estudios realizados con estudiantes de medicina sugieren que la exposición temprana y mantenida a una educación en CP se asocia a actitudes positivas y de mayor satisfacción con los CP^27^. Los estudiantes de enfermería piensan que la formación en CP debería ser un componente esencial de su educación porque contribuyen positivamente a un mayor desarrollo tanto personal como profesional^4^.

La integración completa de cursos de CP en los currículos de pregrado de todos los futuros trabajadores de salud es tanto una obligación amparada por las leyes internacionales como una estrategia educativa basada en la evidencia.

#### A los trabajadores de la salud

Además de pedir educación básica en CP durante el periodo de formación profesional para los médicos y enfermeras, la OMS urge a los Estados Miembros a garantizar formación de nivel intermedio a todos los profesionales sanitarios que tratan habitualmente con pacientes con enfermedades con compromiso vital, y a integrar completamente los CP en todos los niveles asistenciales -especialmente en el nivel comunitario- y a lo largo de toda la trayectoria de la enfermedad avanzada. También se solicita a los Estados Miembros que proporcionen formación especializada a los profesionales sanitarios que están más involucrados en la práctica de los CP^4^. Esto supone que estos profesionales deben recibir una adecuada certificación cuando alcancen las competencias que el título requiera. La educación especializada es de particular importancia en los sitios en los que el rol de los especialistas de CP todavía no ha sido reconocido.

#### A los hospitales y centros de salud

La ciencia médica moderna, desgraciadamente, está cada vez mas basada en la tecnología; se ha centrado tanto en la enfermedad que ha terminado por descuidar el ser humano. El sufrimiento relacionado con la salud se ignora con frecuencia.

La insistencia en intentar tratar la enfermedad, incluso cuando un tratamiento se muestra inútil, causa -además de un sufrimiento físico, social y mental-, dificultades económicas y estrés espiritual. El Papa Francisco en su apelación a los participantes de la asamblea plenaria de la PAV (Aula Clementina, 5 de marzo de 2015) dijo: “Agradezco vuestros esfuerzos científicos y culturales para garantizar que los CP puedan llegar a todos aquellos que los necesitan. Animo a los profesionales y a los estudiantes a especializarse en este tipo de atención, que no tiene menos valor por el hecho de no salvar vidas. Los CP reconocen algo igual de importante: el valor de la persona”^15^.

La Asamblea Mundial de la Salud en su emblemática resolución de 2014^4^ interpeló a todos los Estados Miembros para que integrasen los CP en todos los niveles asistenciales (primario, secundario y terciario) y a lo largo del proceso continuo de cuidado (desde el momento en el que comienza el sufrimiento relacionado con la salud hasta el fallecimiento del paciente, y continuando desde ese momento en forma de apoyo a la familia durante el duelo).

#### A las asociaciones de profesionales de CP

Con frecuencia los pacientes que necesitan CP sufren varias enfermedades simultáneas, pueden estar en su domicilio, en centros de larga estancia, en residencia o en hospitales. Una atención holística de estas situaciones requiere necesariamente equipos multi-disciplinares. Estos equipos pueden pertenecer al sistema nacional de salud, a instituciones religiosas o a organizaciones no gubernamentales^4^.

Los equipos necesitan planificar sus intervenciones en base a las necesidades de los pacientes, ya sean éstos adultos o niños, y de sus familias^28^. Para desarrollar habilidades profesionales y actualizar sus conocimientos, los miembros de los equipos multidisciplinarios cuentan con las guías y recomendaciones realizadas por sociedades y asociaciones de CP, que con frecuencia trabajan conjuntamente con gobiernos, otras agencias de la sociedad civil, donantes o promotores de los CP en el establecimiento del desarrollo de capacidades, provisión de servicios, y la construcción de redes de investigación^29^. De este modo, todos juntos construyen un sistema que alcanza incluso a las comunidades más desfavorecidas desatendidas por los sistemas convencionales de salud^30^.

### Reflexiones para grupos de interés de segundo nivel en la promoción de CP

#### A las organizaciones internacionales

Reconociendo que más del 75% del mundo no tiene acceso a servicios de CP, los Estados Miembros de la OMS adoptaron por unanimidad la Resolución 67/19 en la Asamblea Mundial de la Salud de 2014. En 2015, los Estados Miembros de las Naciones Unidas adoptaron por unanimidad también, la Agenda 2030 del Desarrollo Sostenible con el compromiso de “no dejar atrás a nadie”. No dejar atrás a nadie quiere decir que los Estados Miembros de las Naciones Unidas y las agencias deben colaborar en el desarrollo de políticas y procedimientos integrados, basados en los derechos humanos, para lograr resultados clave de salud pública. Unas políticas de salud pública basadas en los Derechos Humanos consiguen servicios integrados, centrados en la persona; y disponibles para todos los ciudadanos, migrantes, y refugiados de todas las edades en todos los establecimientos sanitarios: domicilio, albergue u hospice, consultas en área rural o urbana, hospital, y establecimientos de larga estancia como residencias o prisiones^3,6,31–38^.

#### A los medios de comunicación

Tanto en el público general como en los profesionales sanitarios existe la idea equivocada de que CP es sinónimo de cuidados al final de la vida^39^. Los CP no son sólo para moribundos. Con este entendimiento viene un imperativo para que los pacientes reciban CP más temprano en su trayectoria de enfermedad^40^. Esto requiere un cambio cultural que vaya desde los médicos a la población general.

#### A las organizaciones filantrópicas y de caridad

Los CP deben integrarse en los sistemas nacionales de salud a nivel mundial. Los gobiernos nacionales no han proporcionado la financiación adecuada para apoyar el desarrollo de los CP, y las organizaciones no gubernamentales, organizaciones profesionales, fundaciones, organizaciones religiosas, organizaciones y fundaciones benéficas, y las agencias del desarrollo, han jugado un papel fundamental en el desarrollo de hospices y CP a nivel internacional y comunitario, proporcionando tanto el apoyo médico como el social. Dada la obligación de los gobiernos de proporcionar cobertura sanitaria universal y un paquete básico de CP, las organizaciones donantes deberían trabajar con los proveedores de servicios de CP para desarrollar sistemas de apoyo social e iniciativas de educación innovadoras^41–44^.

#### A autoridades farmaceúticas

La morfina está recomendada por la OMS como el opioide fuerte de primera linea para el manejo del dolor moderado-severo en cáncer, tanto para adultos como para niños^45–48^. Aunque la morfina está disponible en diversas formulaciones^49^, se recomienda que la prioridad sea la disponibilidad de morfina de liberación inmediata por ser más asequible en precio y por su flexibilidad de uso^50^. Aunque haya otros opioides fuertes novedosos que también debieran estar disponibles, la disponibilidad de éstos otros opioides no es tan importante como la disponibilidad de morfina.

#### A pacientes y grupos de pacientes

Incluso en aquellos países donde los CP están bien desarrollados, falta conocimiento en salud^40^ y se dificulta una intervención temprana de los CP que mejoraría el tratamiento de la enfermedad de base. También, algunos pacientes piensan equivocadamente que tratar síntomas es un modo de acelerar la muerte^51^. Es una incultura sanitaria importante que los pacientes y sus familias no sepan que los CP pueden ser brindados al tiempo que los tratamientos dirigidos contra la enfermedad. Acciones educativas dirigidas a los grupos que piensan así podrían ayudar a disipar mitos existentes sobre unos CP que aceleran la muerte, o que están destinados a pacientes que van a fallecer.

#### A los profesionales del cuidado espiritual

La OMS ha reconocido el cuidado espiritual como un elemento necesario de los CP. El distrés espiritual (sufrimiento espiritual o existencial) ha de ser abordado por todos los miembros del equipo para proporcionar la mayor calidad de vida posible para los pacientes y sus familias, y con el objeto de aliviar su sufrimiento. En varias reuniones internacionales de consenso se han desarrollado definiciones y modelos para detectar y tratar el distrés espiritual en el entorno clínico^52^.

Los líderes religiosos deberían promover la inclusión de cuidados espirituales inter-profesionales en CP, y abogar por la formación de todos los clínicos para proporcionar cuidado espiritual a los pacientes y familias. Al tiempo se debe desarrollar, formar, y ayudar a mantener, una dotación adecuada de personal de capellanía en todos los entornos sanitarios^53,54^.

#### Sociedades y asociaciones profesionales ajenas a los CP

El alivio del dolor y los CP han sido ampliamente reconocidos por muchas instituciones y organizaciones como un derecho humano^4,10,49,55,56^.

#### Farmaceúticos

Con frecuencia los pacientes de CP necesitan tomar multiples medicaciones simultáneamente y, como resultado, se exponen a un riesgo de interacciones farmacológicas que se añaden a los efectos secundarios de los medicamentos esenciales de CP. Los farmaceúticos poseen más conocimientos sobre los medicamentos y sus efectos que ningún otro miembro del equipo sanitario y, por tanto, son los mejor preparados para detectar posibles problemas y hacer las recomendaciones oportunas^57,58^.

## Discusión

En linea con los objetivos de PAL-LIFE, este documento presenta el consenso de 13 expertos de CP de todo el mundo respecto a lo que se ha considerado como las recomendaciones más importantes para 13 grupos de interés claves en la promoción del desarrollo de CP. Algunas de las recomendaciones son aplicables a varios grupos de interés (ej.: la recomendación a las autoridades farmaceúticas sobre la disponibilidad de morfina también debiera dirigirse a legisladores, administradores, productores farmaceúticos, distribuidores y promotores de CP; o la recomendación a las universidades también podría dirigirse a los propios trabajadores sanitarios o a los educadores).

Muchos de las recomendaciones presentadas aquí necesitan un enfoque coordinado. Globalmente, una mayoría de los pacientes mueren con dolor severo sin haber recibido una sola dosis de morfina u otros analgésicos opioides. Para abordar esta trágica situación, es importante armonizar la necesidad de un creciente acceso a opioides para tratamiento del dolor, con tener en cuenta potenciales abusos y efectos adversos. Esta situación demanda un abordaje coordinado entre responsables políticos, universidades, farmaceúticos y sociedades profesionales para poner en marcha medidas de seguridad conforme a los objetivos marcados.

Las recomendaciones en este documento oficial se centran en asuntos cruciales. Sin embargo, para alcanzar situaciones óptimas, se requerirían también otras recomendaciones más amplias y completas (ej.: la recomendación sobre la disponibilidad de la morfina formulada a las autoridades farmaceúticas debería acompañarse de una declaración en la que se clarificase que se necesita más de un opioide barato, ya que hasta un 80% de los pacientes necesitarán rotación opioide en algún momento). El punto crítico reside en que los gobiernos deberían dar los pasos necesarios para garantizar el acceso a los medicamentos de CP incluidos en la lista de medicamentos esenciales de la OMS, entre ellos la morfina como modelo de referencia, así como todos los demás medicamentos de la lista. En el mismo sentido, algunas afirmaciones pueden no capturar con claridad la importancia del cuidado espiritual al mismo nivel que el cuidado físico o el psicosocial. El sufrimiento espiritual, religioso y existencial también debería abordarse, registrarse, monitorizarse y tratarse por parte del equipo de CP.

Una limitación a tener en cuenta en este estudio, es que las recomendaciones están basadas en el consenso de un grupo pequeño de expertos en CP (trece), con la posterior aprobación y respaldo de la PAV. Un grupo mayor pudiera haber aportado más grupos de actores clave en la promoción de CP, y hubiera, por tanto, ampliado el foco de la presente declaración. Por este motivo, el grupo recomienda enérgicamente considerar las recomendaciones ampliamente en su conjunto, así como tener en cuenta las otras 30 recomendaciones adicionales consensuadas por el grupo ad hoc, y que están disponibles en el sitio web de la PAV (www.academyforlife.va).

Este documento representa la Posición Oficial de la PAV en relación a los CP. Cuidar al enfermo ha sido siempre parte de la actividad misionera de la Igesia Católica. La Iglesia se refiere a los CP como “una forma especial de caridad desinteresada que, como tal, ha de ser fomentada” (Catecismo de la Iglesia Católica, n. 2279). El magisterio de la Iglesia Católica ha intervenido en varias ocasiones en los últimos años para enfatizar la dignidad y valor precioso de cada ser humano, también de aquellos afligidos por enfermedades serias o terminales. Recientemente, el Papa Francisco describió los CP como “una expresión de la verdadera actitud humana de cuidar unos de otros, especialmente de aquellos que sufren. Es testimonio de que la persona es siempre preciosa, incluso si ésta padece enfermedad o tiene una edad muy avanzada. […] Por ello, aprecio el compromiso científico y cultural destinado a garantizar los CP a todos aquellos que lo necesiten. Animo a los profesionales y a los estudiantes a especializarse en este tipo de cuidados que no son menos valiosos por el hecho de no salvar vidas. Los CP logran algo igualmente importante: valoran la persona”^15^.

El movimiento cristiano, de acuerdo con las enseñanzas de Jesús de cuidar al indigente, al desfavorecido, y a los pobres, ha desarrollado y construido una amplia red de hospitales, clínicas, y centros de salud por todo el mundo. Los hospitales e instituciones de tipo confesional, desde clínicas locales a centros especializados de investigación, son entornos todos donde CP encajan a la perfección tanto como parte del concepto de cuidado y solidaridad, como un tipo de atención del sistema de salud. En muchos países, independientemente de la fe más profesada, un número sustancial de establecimientos sanitarios están operados por la Iglesia Católica y otras confesiones cristianas. Con tal red, la Iglesia tiene la inmensa oportunidad de aliviar el sufrimiento de millones de pacientes y sus familias.

Este documento de posición oficial puede usarse como un *checklist* para países y regiones para la identificación e implementación de estrategias básicas que mejoren el cuidado de pacientes y familias con necesidades paliativas. También puede servir como el documento base para el desarrollo de una lista mayor de recomendaciones, adaptadas a las organizaciones e instituciones propias de cada grupo de interés, o a los contextos geográficos específicos. Será además de indudable utilidad para la promoción de CP con los gobiernos locales, comunidades religiosas y demás.

En definitiva, este documento enfatiza la responsabilidad de los sistemas de salud e instituciones en el reconocimiento del acceso al alivio del dolor y de los CP como un derecho básico de la persona y de la familia, que es también la responsabilidad de todos los elementos del sistema sanitario. Por ésto, es necesario no sólo reconocer la enfermedad como la ausencia de salud, sino como el bienestar físico, emocional, social y espiritual; cuya optimización pasa por que las medicinas esenciales de CP estén disponibles, por que los gobiernos integren CP en sus planes sanitarios y en la cobertura sanitaria universal, y por el desarrollo de una educación para profesionales y público en general, así como por promover un marco nítido para la implementación de estos cuidados capaces de prevenir el sufrimiento innecesario. El apoyo de organizaciones religiosas y filantrópicas, actores gubernamentales y no gubernamentales y de organizaciones de derechos humanos son necesarios para avanzar en la integración de CP. En resumen, se necesita una respuesta de la sociedad civil.

## Agradecimientos

Esta investigación no ha recibido financiación específica de ninguna agencia financiadora de sectores públicos, comerciales o sin ánimo de lucro. Este trabajo ha sido respaldado en especie por la Academia Pontificia de la Vida (PAV), el Instituto de Cultura y Sociedad de la Universidad de Navarra y por todos los individuos que firman este proyecto.
